# Virome drift in ulcerative colitis patients: faecal microbiota transplantation results in minimal phage engraftment dominated by microviruses

**DOI:** 10.1080/19490976.2025.2499575

**Published:** 2025-05-15

**Authors:** Daan Jansen, Sara Deleu, Clara Caenepeel, Tine Marcelis, Ceren Simsek, Gwen Falony, Kathleen Machiels, João Sabino, Jeroen Raes, Séverine Vermeire, Jelle Matthijnssens

**Affiliations:** a Department of Microbiology, Immunology and Transplantation, Rega Institute, Laboratory of Viral Metagenomics, KU Leuven, Leuven, Belgium; b Translational Research Center for Gastrointestinal Disorders (TARGID), KU Leuven, University Hospitals Leuven, Leuven, Belgium; c Department of Microbiology Immunology and Transplantation, Rega Institute, Laboratory of Molecular Bacteriology, KU Leuven, Leuven, Belgium; d Center for Microbiology, VIB, Leuven, Belgium; e Institute of Medical Microbiology and Hygiene and Research Centre for Immunotherapy (FZI), University Medical Centre of the Johannes Gutenberg-University Mainz, Mainz, Germany

**Keywords:** Ulcerative colitis, fecal microbiota transplants, virome, virome drift, virome community types

## Abstract

Ulcerative colitis (UC) is an inflammatory bowel disease characterized by recurrent colonic inflammation. Standard treatments focus on controlling inflammation but remain ineffective for one-third of patients. This underscores the need for alternative approaches, such as fecal microbiota transplantation (FMT), which transfers healthy donor microbiota to patients. The role of viruses in this process, however, remains underexplored. To address this, we analyzed the gut virome using metagenomic sequencing of enriched viral particles from 320 longitudinal fecal samples of 44 patients enrolled in the RESTORE-UC FMT trial. Patients were treated with FMTs from healthy donors (allogenic, treatment) or themselves (autologous, control). We found that colonic inflammation, both its presence and location, had a greater impact on the gut virome than FMT itself. In autologous FMT patients, the virome was unstable and showed rapid divergence over time, a phenomenon we termed virome drift. In allogenic FMT patients, the virome temporarily shifted toward the healthy donor, lasting up to 5 weeks and primarily driven by microviruses. Notably, two distinct virome configurations were identified and linked to either healthy donors or patients. In conclusion, inflammation strongly affects the gut virome in UC patients, which may lead to instability and obstruct the engraftment of allogeneic FMT.

## Introduction

Ulcerative colitis (UC) is an inflammatory bowel disease characterized by recurrent inflammation in the large intestine, affecting approximately 6.8 million people around the globe.^1,2^ While the exact cause of the disease remains unknown, multiple factors are thought to contribute to its pathophysiology.^3,4^ These factors include environmental triggers, genetic susceptibility, an aberrant immune response and the gut microbiota, all of which interact to fuel intestinal inflammation.^3^ The inflammation is continuous starting in the rectum and gradually extending to more proximal parts of the colon.^4^ Despite extensive research endeavors, no cure is found yet, and current treatment strategies focus on controlling inflammation, alleviating symptoms and inducing remission.^5^ However, remission rates with the approved treatments are ceiling at approximately 30% leaving much room for improvement and alternative treatment strategies.^6^ One promising approach is fecal microbiota transplantation (FMT), a treatment that involves transferring microbiota from healthy donors into the gastrointestinal tract of patients, with the goal of restoring gut dysbiosis and promoting remission.^7–10^

In the context of UC, gut dysbiosis is characterized by an ongoing microbial imbalance in the gastrointestinal tract.^11–15^ This imbalance mainly manifests as a reduced abundance of “Firmicutes” (former bacterial phyla),^13^ an increased abundance of “Proteobacteria”,^14,15^ and is accompanied by a low bacterial diversity and cell count.^12^ As a result, patients consistently show decreased levels of beneficial butyrate-producing bacteria, such as *Faecalibacterium prausnitzii* and *Roseburia homini*,^11^ alongside elevated levels of pro-inflammatory bacteria such as *Escherichia coli*.^16^ Interestingly, the aforementioned dysbiotic pattern corresponds largely to enterotype Bacteroides2,^17^ a microbial constellation which is frequently observed in UC patients, but rarely found in healthy individuals.^12^ While bacterial dysbiosis in UC is quite well-documented, the viral component is less extensively characterized;^18–22^ yet it consistently demonstrates a lower abundance of *Microviridae* phages and a higher abundance of *Caudoviricetes* phages.^18,21^ This viral imbalance is associated with a heightened ability of phages to lysogenize the host (referred to as lysogenic potential),^19,22^ possibly triggered by inflammation, causing prophages to initiate the lytic life cycle.^23^ Finally, viral diversity changes may be a significant factor in this imbalance, but inconsistent results,^19–21^ likely stemming from methodological biases (i.e., genome fragmentation), hinder firm conclusions.^24^

Although the role of the bacterial community in FMT is extensively studied in UC patients,^25–29^ there is limited research regarding the role of the viral community in this process.^30–32^ Few studies indicate that the role of the viral component may have a more significant influence on the efficacy of FMT than previously expected.^30–32^ This finding is not entirely surprising, as intestinal viruses are believed to be as abundant as intestinal bacteria,^33^ and possess the ability to infect and alter the bacterial composition and functional potential.^34^ Building upon this understanding, Gogokhia and colleagues demonstrated that the effectiveness of transplantation was linked to the abundance of “Caudovirales” (former virus order), with patients who respond to FMT exhibiting lower levels of these phages.^30^ Nonetheless, the broader role of the gut virota in the context of UC patients undergoing fecal transplantation has yet to be fully elucidated.^35^

To shed light on this matter, we performed an extensive longitudinal analysis of the gut virota in 44 UC patients who received either autologous (serving as control) or allogenic FMT (serving as treatment).^36^ Autologous FMTs were derived from the patients themselves, while allogenic FMTs were derived from healthy donors. Our goal was to gain a more detailed understanding of the degree of viral engraftment and to outline the factors that influence this process. In addition, we sought to investigate whether certain viral groups are transferred more efficiently. By combining these efforts, we aimed to uncover the conditions most suitable for efficiently transplanting the gut virome.

## Methods

### Ethical approval

The study was approved by the ethical committee of UZ/KU Leuven (reference number: S59525). Participants provided signed informed consent to participate in the study. The design of the study was in accordance with the Declaration of Helsinki and Belgian privacy law.

### Study design

The multicenter randomized clinical trial RESTORE-UC (NCT03110289) enrolled 66 active UC patients who were randomized to receive either autologous (serving as controls) or allogenic (serving as treatment) fecal microbiota transplantation.^36^ The diagnosis of ulcerative colitis (UC) patients was based on clinical, endoscopic and histologic criteria.^37^ Patients were required to exhibit active disease before the start of FMT treatment (baseline) as determined by endoscopic evaluation (total Mayo 4–10, and endoscopic subscore ≥2). Each patients received a sequence of four FMTs, collectively referred to as a “donor batch”, administered at distinct time intervals (baseline and weeks 1, 2 and 3). Notably, all four FMTs within each “donor batch” were consistently derived from the same donor. In this framework, allogenic (healthy) donors were screened based on international consensus guidelines that ensured their overall health through the evaluation of health questionnaires, blood tests and stool tests.^38^ After confirming their health status, allogenic donors underwent another round of screening to identify those individuals with a gut microbiome that was considered favorable (high microbial cell count, and exclusion of Bacteroides2-enterotype and certain pathobionts such as *Veillonella*, *Fusobacterium* and *Escherichia*/*Shigella*). Practically, selected allogenic donors provided fecal samples, which were immediately transported at 4°C at the research institute. Within five hours, the samples were processed in an anaerobic chamber (Whitley A35 Workstation). A minimum of 50 grams was required and homogenized with 500 mL of 0.9% saline solution. Subsequently, the homogenate was filtered using a 5 µm filter (Minisart) to produce the final FMTs. The same procedure was followed for the preparation of autologous FMTs. The primary endpoint was clinical steroid-free remission at week 8 characterized by a total Mayo score ≤2 and Mayo subscores including endoscopy ≤1. Non-responders at week 8 who received autologous FMTs could transition to allogenic FMT as an open-label option. Importantly, the final study cohort for virome analysis comprised 44 UC patients, selected based on the availability of both baseline and week 8 samples. Overall, the trial enrolled fewer patients than initially planned, as the RESTORE-UC study was halted at 66% of its intended inclusions following an interim futility analysis. Multiple factors likely played a role in this negative outcome, with donor selection, FMT preparation and administration being important considerations. However, patient characteristics may have been the most critical factor, as the trial primarily included refractory patients with severe disease. This raises the question whether FMT might achieve greater success in patients with milder forms of the disease. A more detailed overview of the RESTORE-UC clinical trial was previously described by Caenepeel, Deleu and Vazquez Catellanos.^36^

### Metadata

The metadata consisted of clinical parameters, anthropometrics and characteristics of fecal samples and are available in supplementary table S1. Clinical parameters and anthropometrics include serum C-Reactive Protein (CRP) (mg/L), hemoglobin (g/dL), disease location or extent (E1, E2 and E3), endoscopic outcome (endoscopic remission/endoscopic non-remission), age (years), sex (male/female) BMI (kg/m^2^), smoking status (active smoker/nonsmoker), disease duration (years), concomitant UC treatment (steroids, thiopurines, 5-aminosalicylates and/or biologicals), family history of UC (yes/no), extra-intestinal manifestations (yes/no) and FMT treatment profile (donor (−allogenic), donor (−autologous), patient (−allogenic), patient (−autologous), patient (−baseline)). Characteristics of fecal samples included moisture content (%), bacterial cell count, fecal calprotectin (µg/g) and enterotypes (*Prevotella*, *Ruminococcus*, *Bacteroides*1 and *Bacteroides*2). The percentage of mass loss from ±0.2 g frozen aliquots (−80°C) was used to calculate the fecal moisture content after lyophilization. The fCAL ELISA kit (Bühlmann, Schönenbuch, Switzerland) was used for the concentration measurement of fecal calprotectin in frozen samples. Microbial loads were assessed in ±0.2 g frozen (−80°C) aliquots, following the methods described before.^12^

### Sample selection, viral metagenomics and bioinformatic processing

A total of 320 fecal samples from a combined group of patients (*N = 44*) and donors (*N = 31*, including 13 allogenic and 18 autologous donors) were analyzed. Only samples from both a baseline and week 8 timepoint were selected. Among the collected samples, 197 samples were obtained from patients who underwent either allogenic (*N = 21*) or autologous FMTs (*N = 23*). The remaining fecal samples (*n = 123*) were directly collected from the donor FMT material given to the patients, which originated from either the allogenic (samples = 54, individuals = 13) or autologous donors (samples = 69, individuals = 18). Throughout this study, the term “remission” and “non-remission” was used to refer to samples from patients who either responded or did not respond, respectively, based on endoscopic outcomes (week 8).

Fecal samples were prepared with the NetoVIR protocol to purify, enrich and sequence viruses as described in detail before.^39^ A total of 315 samples were successfully prepared and sequenced on a NovaSeq 6000 S2 sequencer (2 × 150, paired end), resulting in an output of 8.90 billion raw reads (equivalent to 1.34 TB). The average number of raw reads per sample was 25.4 million, with a range spanning from 4.43 to 79.2 million. After concluding the sequencing phase, we proceeded with extensive bioinformatic analysis to produce a high-quality dataset, as described in detail before.^22^ In summary, the raw reads were trimmed using trimmomatic v0.39 to remove adapters sequences and low-quality bases.^40^ Next, trimmed reads that aligned with either the reference human genome (hg38, BioProject=PRJNA31257) or contaminome – sequences present in negative controls – were removed to produce a set of high-quality reads using bwa-mem2 v2.0.^41^ These high-quality reads were then *de novo* assembled into long contiguous sequences (contigs) using MetaSPAdes v3.15.1.^42^ To eliminate redundancy, the contigs of all samples were further clustered (95% ANI and 85% coverage) using CheckV’s clustering scripts,^43^ leading to a unique dataset of non-redundant (NR) contigs (*n = 28726*). Additionally, abundance calculations were performed for each sample by mapping (%ANI = 90) the high-quality reads to the set of NR contigs using bwa-mem2 v2.0.^41^ Any contig with a horizontal coverage below the 70% threshold had its abundance value converted to zero. A total of 304 samples were successfully processed and revealed an average number of 4.72 million mapped reads per sample, with a range spanning from 0.0073 to 46.6 million.

### Viral identification and classification

Eukaryotic viruses were identified and classified by aligning NR contigs against extensively annotated NCBI protein and nucleotide databases. Initially, NR contigs were aligned with the conserved NR protein database (August 29, 2022) using CAT v4.6^44^ and DIAMOND v2.0.11^45^ to perform an initial classification. The remaining unclassified NR contigs were then aligned against the nucleotide database (August 29, 2022) using BLASTN v2.11.0 (e-value <1e-10),^46^ refining the viral classification by finding additional matches. The final classification of eukaryotic viruses was determined by identifying the lowest common ancestor for the matches obtained in the previous steps using the ktClassifyBLAST module in KronaTools v2.7.^47^ To ensure accuracy and minimize false positives, an additional examination was conducted on the alignment results with the highest bit score, also known as the top hit. Classifications with an alignment score (AAI/ANI x query coverage) below 0.1 for the top hit were regarded as unclassified. Using these criteria, a total of 34 NR contigs were identified and classified as eukaryotic viruses, with further details available in supplementary table S2.

Prokaryotic viruses (dsDNA, ssDNA or RNA) were identified using Virsorter2 v2.2.3 (–min-score ≥0.5) and the completeness of their genomes was estimated using CheckV v0.8.1.^48^ Those viruses showing a high level of genome completeness, as determined by CheckV’s estimation (≥50% completeness for dsDNA) or meeting the defined criteria for genome sizes (≥3kb for ssDNA/RNA), were classified as “high-quality phages” and selected for further analyses. While RNA phages represent a relatively understudied viral category, recent literature has shown that their genomes typically exceed the 3kb threshold,^49^ except for bi-segmented Picobirnaviruses, which were exempted from the 3kb criterion. High-quality phages were classified (order and class) by integrating the aforementioned homology-based approaches^44–46^ with the classification obtained from Cenote-Taker2.^50^ Furthermore, to enhance the classifications of members of the *Crassvirales*, we conducted a comparative analysis using BLASTN (e-value ≤1e-5, %cov ≥10,000 bp) against a custom nucleotide database, as described earlier.^22^ In order to determine the viral life cycle, the presence or absence of lysogeny-specific genes was examined through Cenote-Taker2, as previously described.^22^ Additionally, for the predictions of bacterial hosts, we employed RaFAH,^51^ an *in silico* host prediction tool, which allowed us to make predictions at both the genus (with a minimum cutoff of ≥ 0.50) and phyla taxon (with a minimum cutoff of ≥ 0.14). Phyla-level host prediction identified 84.1% of hosts, while genus-level prediction identified only 24.1%. Given the higher accuracy at the phyla level, we chose to primarily rely on phyla-level predictions for further analyses. Finally, NR-contigs that could not be identified as either eukaryotic or prokaryotic viruses were also aligned against the NCBI protein and nucleotide database. Subsequently, they were categorized as bacterial, other (encompassing archaea, protozoa and eukaryota) and dark matter (unannotated).

### Viral intra and inter-individual variation

In accordance with *Nayfach* and colleagues,^52^ the NR phage contigs were grouped into genus-like clusters (resembling phage genera), by employing a clustering approach based on a combination of pairwise average amino acid identity and gene sharing. Virome intra-individual variation, commonly known as alpha-diversity, was calculated using Shannon diversity on the genus-like abundance table using phyloseq.^53^ Virome inter-individual variation, commonly known as beta-diversity, was calculated using Bray-Curtis dissimilarity. Principal coordinates analysis was used to visualize the dissimilarity matrix obtained after Hellinger transformation of the genus-like abundance table. To analyze the explanatory effect of metadata variables (i.e., covariates) on the relative virome profiles (genus-like level, Hellinger transformation), we utilized univariate and multivariate stepwise distance-based redundancy analysis (dbRDA) using the capscale function implemented in the vegan package.^54^ Metadata variables that revealed a significant contribution to virome variation in the univariate dbRDA, were subsequently integrated into the multivariate dbRDA using a forward selection model with the *ordiR2step* function in vegan.

### Donor virome transplantation

The degree of virome engraftment within the intestinal tract of UC patients undergoing FMT was determined by calculating the Bray-Curtis dissimilarity index and the percentage of shared donor genera between patients and their respective donor batch. The Bray-Curtis dissimilarity index was calculated between the relative virome profiles (genus-like level, Hellinger transformation) of each patient sample and their corresponding donor batch. To determine the effect of FMTs, we compared samples collected after FMT (w4, w8, w12, m6, and m12) with baseline samples (w0), where an effective FMT would manifest as a reduced dissimilarity over time. The percentage of shared donor genera was computed as the fraction of genera present in the corresponding donor batch that were simultaneously identified in individual patient samples. Moving forward, the viral genera that were successfully engrafted within the intestinal tract of UC patients were identified at week 4 (1 week post FMTs) and week 8 (5 weeks post FMTs). The samples underwent an initial rarefaction to standardize the read count. Abundance changes were calculated for genera, exclusively present in the donor batch, between the patient’s baseline and post-FMT (week 4 and week 8) timepoints. Genera with a 100% or more abundance increase (expressed as a log2 fold change ≥ 1), corresponding to the 86.68th percentile of allogeneic phage transplants, were considered successfully transplanted, as they reflect the most substantial engraftment events. Finally, the viral NR-contigs that were successfully engrafted within the intestinal tract of UC patients were identified using the same method at week 4 and week 8. Importantly, since phage transplantation is calculated based on abundance increases, and given that external factors can influence these abundances, it is important to add the nuance that some unknown factors (not included in the trial metadata) could potentially affect the calculations

### Viral community types

Viral community typing was performed on the genus-level virome profiles (i.e., genus-like clusters with Hellinger transformation) using Dirichlet multinomial mixtures (DMM) implemented in the DirichletMultinomial package.^55^ This approach has been used by our group as well as other, and represents an adaptation of “enterotyping”, a technique commonly used in microbiome research.^22,56,57^ To enhance accuracy, a 1% prevalence threshold for taxa was included. The optimal number of clusters or Dirichlet components were determined to be two based on the Bayesian Information Criterion (BIC) score (*n = 301*, Supplementary Figure S8). The mean probability of clustering assignment was 0.996 (median = 1.00; Supplementary Table S13).

### Statistics

Statistical analyses followed a two-sided, non-parametric approach with a predefined significance level of *p* < 0.05. Multiple testing correction was conducted using the Benjamini-Hochberg (BH) method, with significance defined as AdjP < 0.05. All the statistical analyses were performed in R^58^ using packages such as vegan,^54^ phyloseq,^53^ lme4^59^ and stats. Effect sizes for Wilcoxon ($r=Z/\sqrt{N}$) and Chi-squared statistics ($r=\sqrt{\chi^{2}}/N$) were computed using the rstatix package. Paired statistics were applied when suitable, and (generalized) linear mixed-effect models (LMM/GLMM) were used to account for the biological dependency of samples from multiple timepoints within the same individual. Patient identifier was included as the grouping variable (random effect) in the latter models.

### Code availability

Bioinformatic processing of raw reads was conducted by the Virome Paired-End Reads (ViPER) pipeline v1.0 available at (https://github.com/Matthijnssenslab/ViPER). All the data to perform virome analyses and ensure the reproducibility of the code can be found at https://github.com/Matthijnssenslab/IBDVirome/tree/main/IBDFMT.

### Data availability

The quality-controlled reads have been submitted to the NCBI Sequence Read Archive and can be accessed with the BioProject accession number PRJNA984221.

## Results

### Characterizing the gut virome in ulcerative colitis patients undergoing fecal microbiota transplantation: phage dominance and occasional plant viruses

The gut virome was characterized of ulcerative colitis patients enrolled in a multicenter randomized sham-controlled clinical trial called RESTORE-UC (Methods).^36^ Patients received a series of four fecal microbiota transplantations, either from a healthy donor (*N* = 21, allogenic FMT) or using their own stool (*N* = 23, autologous FMT). Most quality-controlled sequencing reads were found to be of viral origin (viral = 67.8%, bacterial = 24.8%, other = 4.6%, dark matter = 2.9%). This indicated that the identified viral fraction served as a reliable representation of the gut virome (Supplementary Figure S2a), a key prerequisite for subsequent virome analyses. Closer examination of the gut virome revealed a predominance of phages alongside a minor presence of eukaryotic viruses, consistent with prior IBD research.^60^ The eukaryotic viruses were found in up to 30.6% of fecal samples, accounting for only 0.7% of the quality-controlled viral reads (Supplementary Figure S2b). Notably, a significant portion of these viruses were plant viruses, likely obtained through dietary consumption and only passing through the human gut (Supplementary Figure S3; Supplementary Table S2). Among these, the most common ones were Pepper mild mottle virus (*Alphaflexiviridae*) responsible for infecting bell peppers, and Pepino mosaic virus (*Virgaviridae*) known to cause tomato infections. Both species were detected in 10.2% and 12.2% of the fecal samples, respectively (Supplementary Table S3). In contrast, phages were ubiquitously present in all fecal samples, accounting for 99.3% of the quality-controlled viral reads (Supplementary Figure S2b). The three most abundant phage orders at the read level were unclassified *Caudoviricetes* (43.7%, tailed dsDNA), *Petitvirales* (43.2%, spherical ssDNA microviruses) and *Crassvirales* (8.83%, tailed dsDNA) (Supplementary Figure S4a). Within each of these orders, the median length of the phage genomes consistently matched the length reported in the literature, implying a reliable phage classification at a high taxonomic level (Supplementary Figure S4b). The phage orders also showed a distinct affinity to particular predicted bacterial host phyla (Supplementary Figure S5). For instance, unclassified *Caudoviricetes* phages are predicted to preferentially infect Bacillota (i.e., “Firmicutes”; 55.5%), while *Petitvirales* favored Pseudomonadota (i.e., “Proteobacteria”; 35.3%) and *Crassvirales* are almost exclusively predicted to infect members of the Bacteroidota (i.e., “Bacteroidetes”; 96.1%).

### A substantial influence of individuality and treatment profiles on the gut virome

**Figure 1.**
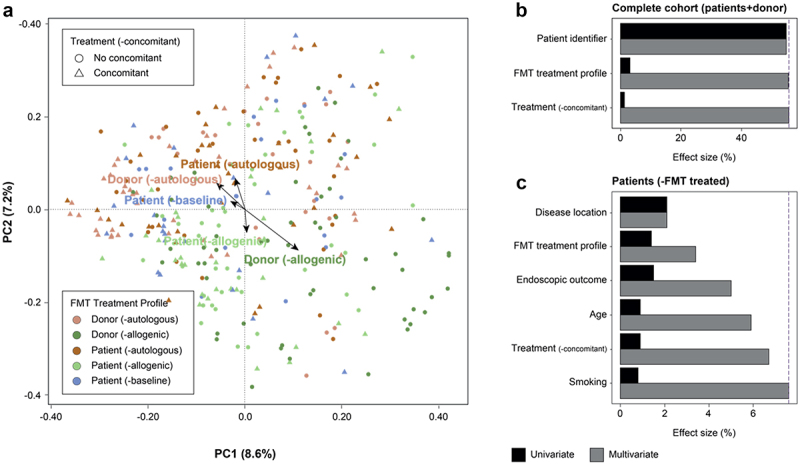
Virome covariates of the UC cohort undergoing fecal microbiota transplantation. (a), Principal coordinate analysis of inter-individual differences in the relative virome profiles (genus-like level, Bray-Curtis dissimilarity) of the complete UC cohort (*n = 301*, biologically dependent samples, coloured by FMT treatment profile). The arrows on the plot represent effect sizes of significant virome covariates in the multivariate model in panel B. (b,c), Clinical metadata correlating to the relative virome profiles in the complete (*n = 301*, Patients+Donors) and FMT treated (*n = 139*, patients) UC cohort (dbRDA, genus-like level, Bray-Curtis dissimilarity), respectively. The effect sizes of correlating variables are calculated using either univariate (coloured in black) or multivariate (coloured in grey) analysis. The multivariate contribution in the latter analysis is indicated by a vertically dashed line and is 7.56%. Correction for multiple testing was performed, when appropriate, with the Benjamini-Hochberg method and significant associations were identified based on AdjP < 0.05. The aforementioned analyses include patient identifier as a grouping variable to account for the biological dependencies among multiple measurements within each patient. Abbreviations: ulcerative colitis (UC) and fecal microbiota transplant (FMT).

Having deepened our understanding of the virome composition, the next step was to explore which factors could affect this composition. To identify relevant factors, we performed a distance-based redundancy analysis (dbRDA) on the virome composition of the complete UC cohort (Patients+Donors; Supplementary Table S4). First of all, we found individuality (the unique patient characteristics, as identified by patient ID) as the most prominent explanatory factor (*n = 301*, multivariate dbRDA, genus-like group, R_2_ = 54.9%, AdjP = 0.002; Figure 1b), a well-established covariate in prior research.^60,61^ This observation underscored the substantial virome variation among different individuals. Besides individuality, it became evident that the nature of the provided treatment (both FMTs and concomitant (other) treatments) had an explanatory effect on the virome composition of the patients, albeit a relatively modest one (*n = 301*, multivariate dbRDA, genus-like group, FMT profile R_2_ = 0.6%, Concomitant treatment R_2_ = 0.2%, AdjP < 0.05; Figure 1a,b).

### Disease location has the largest effect on the gut virome composition in ulcerative colitis, overshadowing the impact of fecal microbiota transplants

In order to assess the impact of environmental factors on the gut virome, we performed a second dbRDA analysis on the UC cohort (excluding donor samples). Beginning with FMT-untreated patients (baseline samples), our study sought to understand the factors influencing the baseline composition (Supplementary Table S5). Initially, we started with 44 patients, but 2 were excluded due to not reaching the viral quality thresholds, resulting in a final sample size of 42 (Extended Data Methods). Fecal calprotectin was identified as the sole significant factor associated with the baseline composition (*n = 42*, univariate dbRDA, genus-like group, R_2_ = 2.43%, AdjP = 0.0160). This association was consistent with the fact that all untreated patients exhibited active inflammation, and fecal calprotectin levels are established markers of neutrophil migration triggered by colonic inflammation.^62^ Additionally, the endoscopic outcome does not explain interindividual variation in virome composition (*n = 42*, univariate dbRDA, genus-like group, AdjP = 0.568). This indicated that the notion of using the virome as a predictive tool for endoscopic outcome (remission/non-remission), as suggested in earlier research,^31^ could not be confirmed. However, it is important to note that the small sample size (*n = 42*) might have obscured potential associations that could have become apparent in larger datasets.Moving on to FMT-treated patients (post-interventional samples), our study aimed to evaluate the effect of the administered FMT treatment on the gut virome composition (Supplementary Table S6). To our surprise, both the presence and the location (or extent) of inflammation held a greater explanatory power than the fecal transplantation (*n = 139*, univariate dbRDA, genus-like group, Disease location R_2_ = 2.07%, Endoscopic outcome = 1.46%, FMT treatment profile R_2_ = 1.27%, AdjP < 0.05; Figure 1c). This pointed to the central role of inflammation in determining the gut virome composition, while suggesting a potentially limited or inefficient transfer of viruses from the donor to the patients. In line with earlier findings,^63^ the age and smoking habits of the patients showed only a minor contribution to the gut virome variation (*n = 139*, multivariate dbRDA, genus-like group, Age R_2_ = 0.890%, Smoking = 0.830%, AdjP < 0.05; Figure 1c). Moreover, in these analyses, the choice of FMT treatment (autologous or allogenic) did not show a significant association with any of the previously mentioned covariates, implying they did not introduce confounding effects (*n = 139*, generalized linear mixed-effect model (GLMM), AdjP > 0.05; Supplementary Table S7). Taken together, these findings suggested that the administered FMT treatment, along with the presence and location of inflammation, influenced the gut viral profile of UC patients.

### A longitudinal virome drift in UC patients receiving autologous FMT

**Figure 2.**
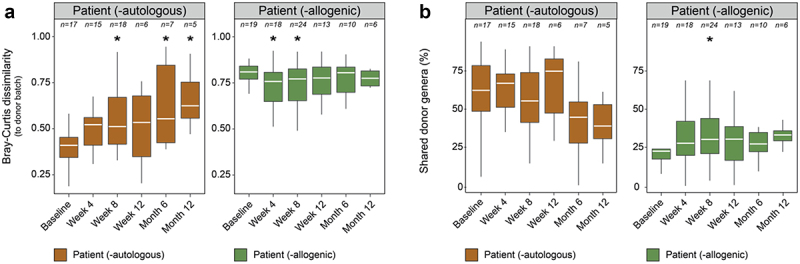
Donor virome engraftments in the complete UC cohort. (a), Boxplot of differences in the relative virome profiles (genus-like level, Hellinger transformation) between patients and their corresponding autologous or allogenic donor sample (LMM, AdjP < 0.5) at different timepoints. (b), Boxplot of the percentage of shared donor taxa between patients and their corresponding donor sample (LMM, AdjP < 0.5) at different timepoints. Shared donor taxa were defined as those genera present in the corresponding donor batch that were simultaneously identified in individual patient samples. Significant associations were determined after multiple testing (Benjamini-Hochberg method) by comparing each post-FMT to baseline timepoint and are represented by an asterisk (*). The aforementioned analyses include patient identifier as a grouping variable to account for the biological dependencies among multiple measurements within each patient. Abbreviations: ulcerative colitis (UC) and linear mixed-effect model (LMM).

Having established the link between the gut virome and the administered FMT, our next goal was to investigate the long-term dynamics of the gut virome in UC patients receiving autologous FMT, thereby creating a crucial baseline for the interpretation of further transplantation analyses. In doing so, we determined the virome similarity of the patients relative to their own donor sample over an extended period (up to 1 year). Although we expected to see a transition toward the donor virome (ie., decrease in Bray-Curtis dissimilarity) immediately after transplantation (week 4- or 1-week post-transplantation), contrary to this expectation, a trend of increasing viral divergence was observed (Figure 2a, left). This trend of a rather rapid divergence of the patients’ gut virome from their corresponding donor virome over time, resulted in a significantly higher Bray-Curtis dissimilarity by week 8, as compared to the baseline-donor dissimilarity (*n = 18*, linear mixed-effect model (LMM), genus-like group, AdjP = 0.0327; Figure 2a, left; Supplementary Table S8). Remarkably, the trend remained significant even with small sample sizes for up to one year (*n = 5*, LMM, genus-like group, AdjP = 0.0327; Supplementary Table S8). Studies on healthy individuals have described long-term stability of the gut virome, whereas our findings in UC patients reveal a shift in virome composition over time.^64^ We termed this phenomenon “virome drift”, a measure of instability that is likely fueled by ongoing inflammation. Interestingly, this phenomenon is reflected in the pronounced dissimilarity between patients’ baseline and donation samples, taken one week to one month prior to the initial transplantation (Figure 2b, left). Apart from our focus on dissimilarity indices, we also determined the presence of donor taxa (approximately genus-level) in the patients’ gut virome over time (Figure 2b, left; Supplementary Table S9). The results showed a high percentage of shared donor taxa that remained stable over time, indicating that these viral genera can persist for at least one year in the gut of UC patients. However, when focusing on relative virome changes in individual UC patients, substantial variations among the viral genera became evident (Supplementary Figure S6). These variations persisted even at a taxonomic level as high as “phage order” over extended periods of time. Altogether, these results revealed a long-term viral instability, or virome drift, in the gut virome of UC patients, mainly characterized by changes in viral abundance profiles.

### Ulcerative colitis patients receiving allogenic FMT, without correlation to clinical outcome, reveal a partial transfer of donor viruses

**Figure 3.**
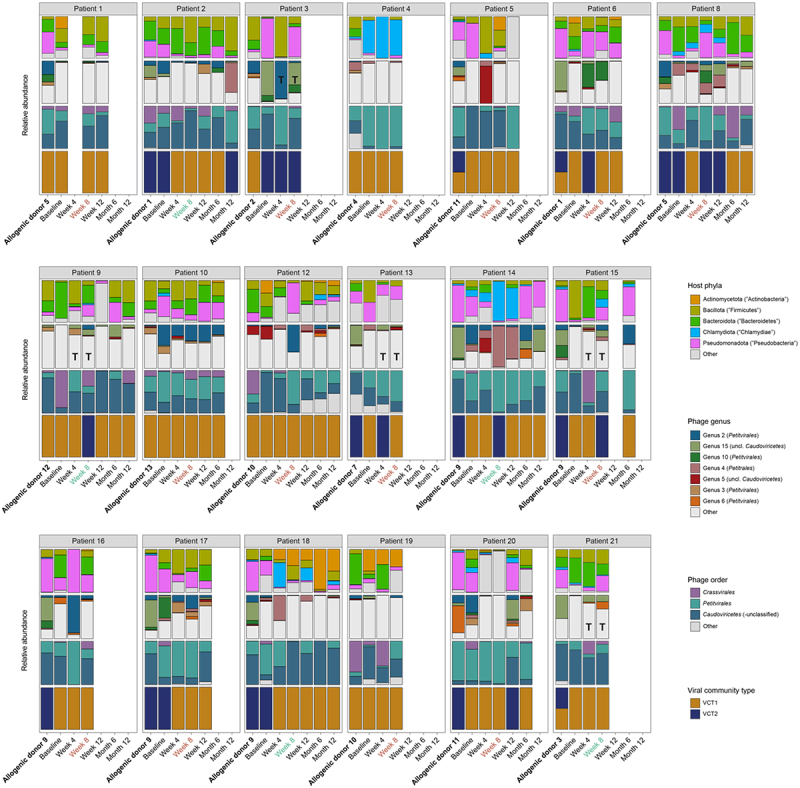
Summary of individual UC patients undergoing allogenic fecal microbiota transplants. Barplot showing the relative abundance of major (≥1% of reads) *in silico* predicted hosts, phage genera (≥15% prevalent), phage classes and viral community types, along a longitudinal axis for each UC patient undergoing healthy donor FMT. Each patient received a sequence of four FMTs, collectively referred to as “Allogenic donor x” (in bold), and were consistently derived from the same donor. Patients are shown who had available samples from the healthy donor, baseline (week 0) and week 8. The endoscopic outcomes at week 8 are represented as either remission (green) or non-remission (red). Patients who exhibit transplanted donor genera at both week 4 or week 8 are indicated by the letter “T”. Abbreviation: fecal microbiota transplantation (FMT) and ulcerative colitis (UC).

Once the baseline dynamics of the gut virome in UC patients had been established, the next step was to explore the long-term virome dynamics in patients starting allogenic FMT. In doing so, we observed a subtle shift of the patients’ gut virome toward the administered donor virome (Figure 2a, right). However, this transition was only partial, demonstrated by a small, yet significant decrease in Bray-Curtis dissimilarity by week 4 (1-week post-FMT, *n = 18*, LMM, genus-like group, AdjP = 0.0232; Supplementary Table S8). This pattern continued until week 8, indicating a short-term retention of donor viruses within the patient’s gut (*n = 24*, LMM, genus-like group, AdjP = 0.0295; Figure 2a, right; Supplementary Table S8). At this timepoint, we also observed a significantly higher fraction of shared donor genera in the gut of allogenic FMT-treated patients (*n = 24*, LMM, genus-like group, AdjP = 0.0249; Figure 2b, right; Supplementary Table S9). Nevertheless, even though there were some resemblances between the gut viromes of patients and donors, our findings did not reveal any difference in virome similarity between patients who achieved remission and those who did not (LMM, genus-like group, AdjP > 0.05; Supplementary Figure S7; Supplementary Table S10). When considering individual UC patients, the majority did not show a notable transition toward the donor virome at a low taxonomic resolution (genus-like). However, for certain patients; including patient 3, patient 9, patient 13, patent 15 and patient 21, a (minor) shift toward the donor virome was observable (Figure 3). Collectively, these findings suggested a partial and temporary transplantation of donor viruses in allogenic FMT-treated patients, supported by both the increased similarity in viral abundance profiles and the closer resemblance of viral genera, detected shortly after transplantation.

### Predominance of microviruses in short-term engraftment of donor bacteriophages

**Figure 4.**
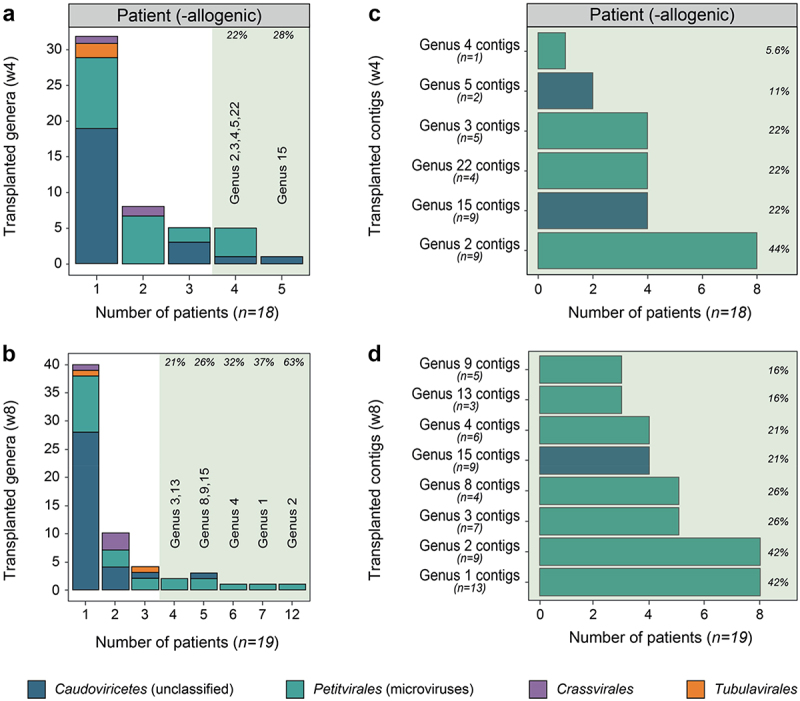
Transplanted donor phages in the complete UC cohort. (a), Barplot of successfully engrafted donor genera (abundance increase ≥ 100%) at week 4 (1 week post-FMT). (b), Barplot of successfully engrafted donor genera (abundance increase ≥ 100%) at week 8 (5 weeks post-FMT). The shaded green section on the plot denotes viral genera transplanted in more than 20% of the patients. (c), Barplot of successfully engrafted donor contigs present within each highly transplanted genus (abundance increase ≥ 100%) at week 4 (1 week post-FMT). (d), Barplot of successfully engrafted donor contigs present within each highly transplanted genus (abundance increase ≥ 100%) at week 8 (5 weeks post-FMT). Abbreviations: ulcerative colitis (UC) and fecal microbiota transplant (FMT).

Our next goal was to conduct a more detailed analysis and identify the particular donor genera that were successfully transplanted. Donor genera were considered transplanted when their abundance increased by 100% or more between the patient’s baseline and post-FMT (week 4 and 8) timepoints (Extended Data Methods). Based on that, 51 donor genera were found to be transplanted by week 4, which rose to 62 genera by week 8, signifying a continuous viral colonization of the gut of UC patients after transplantation (Figure 4a,b). Certain donor genera demonstrated a greater transplantation efficiency, as they were transplanted in 20% or more of UC patients, accounting for 6 genera at week 4 and expanding to 8 genera by week 8 (Figure 4a-d; Supplementary Table S11). It is worth mentioning that 80% of these commonly transplanted genera were classified as *Petitvirales*, namely microviruses, whereas the remaining 20% were classified as *Caudoviricetes* phages (Supplementary Table S11). Interestingly, among the most transplanted genera at both timepoints, Genus 15 and Genus 2, were predicted to infect Pseudomonadota (Supplementary Table S11), a bacterial phylum commonly found in UC patients.^15^ To offer additional evidence of the temporal transplantation of phages, our focus shifted from donor genera to individual donor phage contigs. By week 4, we identified 99 transplanted phage contigs, averaging 5.5 phages per patient. This number increased to 125 by week 8, with an average of 6.6 phages per patient. Most of these phages (93 at week 4 and 116 at week 8) were transplanted only once, making them unique to each donor-patient pair. Additionally, we confirmed the successful transplantation of numerous individual phages from the most commonly transplanted genera (Figure 4c,d). Altogether, the results revealed a short-term engraftment of donor bacteriophages in the gut of UC patients, validated by the transfer of individual donor phages, most of which were classified as members of the *Petitvirales*.

### No evidence of donor virome community transmission in UC patients

**Figure 5.**
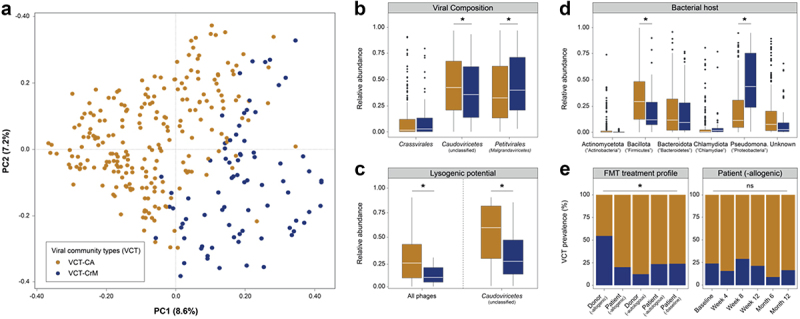
Viral community types of the complete UC cohort. (a), Principal coordinate analysis of inter-individual difference in the relative virome profiles (genus-like level, Bray-Curtis dissimilarity) of the complete UC cohort (*n = 301*, biologically dependent samples, coloured by VCT). (b), Boxplot showing relative abundance of key phage classes categorized according to VCT (*n = 301*, LMM, AdjP < 0.5). (c), Boxplot showing relative abundance of lysogenic potential of all discovered phages (left) and unclassified *Caudoviricetes* phages (right) categorized according to their VCT (*n = 301*, LMM, AdjP < 0.5). (d), Boxplot showing relative abundance of major predicted bacterial hosts (≥1% of reads) categorized according to their VCT (*n = 301*, LMM, AdjP < 0.5). (e), left, Barplot showing VCT prevalence in complete UC cohort categorized according to FMT treatment profile (*n = 301*, GLMM, AdjP < 0.5). (e), right, Barplot showing the longitudinal prevalence of VCT in the allogenic FMT-treated cohort (*n = 99*, GLMM, AdjP < 0.5). Multiple testing adjustment (Benjamini-Hochberg method) was performed and significant associations (AdjP <0.05) are represented by an asterisk (*). The aforementioned analyses include patient identifier as a grouping variable to account for the biological dependencies among multiple measurements within each patient. Abbreviations: ulcerative colitis (UC), viral community types (VCT), linear mixed-effect model (LMM) and generalized linear mixed-effect model (GLMM) and non-significant (ns).

To assess the efficacy of viral transplantations within a virome community context, rather than focusing solely on individually transplanted viral genera or contigs, we conducted viral community typing (See methods). Through this analysis, we identified the presence of 2 virome communities that exhibited characteristics similar to a prior IBD studies (*n = 301*, genus-like group, Bray-Curtis dissimilarity; Figure 5a; Supplementary Figure S8a; Supplementary Table S12).^22,57^ The virome configurations were termed viral community type CA (VCT CA) and viral community type CrM (VCT CrM), each marked by a significantly higher relative abundance of either *Caudoviricetes* (−unclassified) or *Malgrandaviricetes* phages (*n = 301*, LMM, AdjP < 0.05; Figure 5b; Supplementary Figure S8b; Supplementary Table S13). Of note, a more detailed and reproducible labeling of the virome configurations based on influential phage genera would be desirable but remains unattainable at this moment due to the absence of a well-defined phage taxonomy for the majority of identified phage genomes. This limitation was evident when considering differentially abundant phages genera between VCT CA (Genus 5) and VCT CrM (Genus 2, Genus 15, Genus 10 and Genus 4), which could only be reliably classified at a higher taxonomic level (*n = 301*, LMM, AdjP < 0.05; Supplementary Figure S8b; Supplementary Table S13,S14). Having established the composition of the community types, the lysogenic potential was investigated and we observed a significantly higher lysogenic capacity among phages in VCT CA (*n = 301*, LMM, AdjP = 2.43e-05; Figure 5c, left; Supplementary Table 13). At first sight, this observation appeared self-evident, as VCT CA was mostly defined by *Caudoviricetes* (−unclassified) phages, often associated with lysogeny. However, even when solely considering the *Caudoviricetes* (−unclassified) phages, a higher lysogenic potential was noted compared to VCT CrM (*n = 301*, LMM, AdjP = 2.79e-05; Figure 5c, right; Supplementary Table S13). Next, to investigate the virome configurations within a broader microbiome context, *in silico* host predictions were conducted. In this respect, a significantly higher relative abundance of Bacillota-infecting phages was found in VCT CA (*n = 301*, LMM, AdjP = 7.33e-03; Figure 5d; Supplementary Table S13). This observation, when considered together with the previous findings, suggested that *Caudoviricetes* (−unclassified) phages in VCT CA were predominantly induced from Bacillota (Figure 5d; Supplementary Figure S5). Additionally, this induction seemed to be associated with ongoing inflammation, as a significantly higher prevalence of VCT CA was detected in UC patients compared to the healthy donors (*n = 301*, GLMM, AdjP = 0.0182; Figure 5e, left; Supplementary Table S15). In contrast, VCT CrM showed an association with primarily lytic phages infecting Pseudomonadota, specifically *Escherichia*, and was mainly found in healthy donors (*n = 301*, LMM, AdjP = 1.73e-07; Figure 5d; Supplementary Figure S9; Supplementary Table S13). This observation was notable, as Pseudomonadota were typically linked to UC patients,^15^ hinting at an inversed relationship between these phages and their hosts. Lastly, irrespective of endoscopic outcome, there were no consistent longitudinal changes in the prevalence of either viral community type among allogenic FMT-treated patients (LMM, AdjP > 0.05; Figure 5e, right; Supplementary Table S15). This suggests that virome communities were not systemically transmitted from the donor to the patients (Figure 3).

## Discussion

To our knowledge, this study marks one of the most comprehensive longitudinal descriptive analyses of the gut virota in UC patients undergoing fecal microbiota transplantation. We reported a eukaryotic virome present in a minority of samples, primarily composed of plant viruses (Supplementary Figure S3), consistent with earlier research.^22,60^ These viruses are likely acquired through dietary consumption and pass through the human gut. This, coupled with the rare occurrence of human-infecting viruses, attributes a low importance to eukaryotic viruses in disease pathology and treatment. Importantly, while we consider these viruses unlikely to have a substantial impact, there is a possibility that eukaryotic viruses, due to their low abundance, may not be detected with our current methodologies. Conversely, in this study, we unraveled an abundantly present phageome, more responsive to localized intestinal inflammation compared to the administered fecal transplants (Figure 1c). This underscores the central role of inflammation along with an inefficient transfer of viruses from the donor to the patients. Inflammation was likewise observed in the autologous FMT-treated patients and may have contributed to virome drift (Figure 2a). This drift contrasted sharply with previous research on healthy individuals, which indicated a long-term stability of the gut virome.^64^ It also points to considerable intrapersonal variation, complementing the recognized interpersonal differences within the virome.^60^ A notable limitation in this study arises from comparing virome studies instead of including a longitudinal dataset of healthy individuals. We recognize that incorporating such a dataset would have substantially strengthened the concept of virome drift in the present study. An additional limitation is the relatively small number of patients in each group — 21 in the allogenic FMT group and 23 in the autologous FMT group – which may limit the generalizability of our findings. While our cohort was densely sampled over time, the limited patient size, a consequence of the early termination of the clinical trial following a negative interim analysis, does restrict the power of the study.^36^

The factors contributing to an ineffective viral transfer are likely multifaceted.^36^ One explanation may be practical limitations associated with the preparation and application of FMT. The FMTs were prepared and administered as rectal enemas, with a recommended retention time of 30 minutes, following established guidelines.^38^ Variability in retention time may have introduced some bias, but data on this was not collected. Another hypothesis we brought forward to explain the difficulties of transplanting donor virota in allogenic FMT-treated patients (Figure 2a,b) involves the impact of the inflammatory environment, building on the concept of the “inflammatory positive feedback loop”.^24^ In essence, persistent colonic inflammation stimulates enterocytes to generate stressors such as reactive oxygen species.^65^ These stressors, in turn, activate the bacterial stress response, known as the SOS response.^66^ This process triggers phage induction, initiating the lytic life cycle and leads to the lysis of the bacterial host.^34^ Subsequently, as a result of increased bacterial breakdown, more pathogen-associated molecular patterns are released into the gastrointestinal tract, including lipopolysaccharide, which stimulate the enterocyte receptors.^67^ To perpetuate the positive feedback loop, enterocytes are incited to produce more stressors, further promoting phage induction and bacterial lysis.^68^ Thus, the inflammatory feedback loop implies that only microbes that are well-adapted to an inflammatory environment can thrive and persist over time. Consistent with this proposition, we showed that, not the entire donor virome community (Figure 5e), but a specific and limited number of donor viruses were successfully engrafted (Figures 2a,b, 4a–d) to the gut of allogenic FMT-treated patients. Further investigation of these viruses revealed that a considerable portion could be classified as microviruses (Figure 4c,d), as previously also demonstrated by Fujimoto and colleagues.^69^ We speculate that the preference of microviruses for infecting Pseudomonadota (Supplementary Figure S5; Supplementary Table S11), a bacterial phylum abundantly present in IBD,^14^ along with their lytic nature, may be important factors facilitating their transmission to UC patients. Nevertheless, the limited phage engraftment observed in the present study suggests that most transplanted donor phages and their bacterial hosts are not well-adapted to the inflammatory conditions in the intestines of UC patients. It is important to note that a complete understanding of the intestinal micro-ecosystem, transplantation efficiency and the phenomenon of virome drift herein would require the integration of bacterial data, something that was beyond the scope of this manuscript.

One might argue that our viral enrichment protocol employed a whole transcriptome amplification (WTA2) kit, potentially introducing bias toward viruses with circular single-stranded genomes, particularly microviruses. However, this concern is unfounded for two key reasons: (1) WTA2 includes a minimal multiple displacement amplification (MDA) step, followed by conventional PCR without MDA; and (2) the protocol was validated using a diverse mock virome containing both linear and circular genomes, demonstrating no evidence of such bias. Instead, the results showed a strong correlation between mapped reads and viral copy numbers.^39^ In addition, a recent study by Haagmans and colleagues found no MDA bias when comparing WTA2 and SISPA amplification methods.^60,70^ That said, a broader limitation of virome studies lies in the compositional nature of the data, which portrays relative proportions or compositions of viral taxa rather than absolute counts. As increases in the abundance of certain taxa are inevitably linked to decreases in others, identifying viruses truly associated to ulcerative colitis or FMT intervention becomes challenging, emphasizing cautious interpretation the results.^71^ Nonetheless, we abstained from using previously suggested compositional normalization techniques (eg., centered log-ratio) as they depend on pseudocounts and substantial pre-filtering thresholds, resulting in considerable distortion of the original data structure.^72^

To conclude, our study revealed a substantial impact of colonic inflammation on the viral profile in UC patients receiving fecal microbiota transplants. We identified a lasting viral instability, associated with the observed inflammation, which could potentially account for the partial engraftment of donor viruses in allogenic FMT-treated patients, and ultimately, negative trial outcome of RESTORE-UC.^36^ Based on this, we hypothesize that the simultaneous use of effective anti-inflammatory drugs and fecal microbiota transplants could establish a more conducive environment for the efficient transplantation of donor viruses and promote intestinal eubiosis.

## Supplementary Material

Supplemental Material

## Data Availability

The metadata encompassing clinical parameters, anthropometrics, and fecal sample characteristics, can be found in Supplementary Table S1. The quality-controlled reads have been deposited into the NCBI Sequence Read Archive and are accessible via the BioProject accession number PRJNA984221. The ViPER (Virome Paired-End Reads) pipeline version 1.0 was utilized to perform initial processing on the raw paired-end reads and can be freely accessed at https://github.com/Matthijnssenslab/ViPER. All the data to perform virome analyses and ensure the reproducibility of the code can be found at https://github.com/Matthijnssenslab/IBDVirome/tree/main/IBDFMT.

## Patients and public involvement statement

The Leuven IBD research group (including Prof. Séverine Vermeire and other coauthors of TARGID) write a yearly newsletter for all participants of their ongoing CCARE Biobank and related research. In this newsletter, the yearly progress is explained and elaborated future plans are discussed. Both researchers and clinicians actively participate in a yearly meeting organized for patients and their family members with the help of the Flemish Crohn’s and Colitis patient association (CCV). Articles are written regularly for the quarterly magazine of the CCV, in which the topic on results of this research project will also be distributed through all of these channels.
