# Supplementary material for: Virome drift in ulcerative colitis patients: faecal microbiota transplantation results in minimal phage engraftment dominated by microviruses
Source: Gut Microbes. 2025 May 15;17(1):2499575. doi: 10.1080/19490976.2025.2499575 (PMC12087655; doi:10.1080/19490976.2025.2499575)
Supplement: Supplemental Material [file KGMI_A_2499575_SM1113.zip › Main_Document__3__Supp.docx]

**VIROME DRIFT IN ULCERATIVE COLITIS PATIENTS: FAECAL MICROBIOTA TRANSPLANTATION RESULTS IN MINIMAL PHAGE ENGRAFTMENT DOMINATED BY MICROVIRUSES**

**EXTENDED DATA FIGURES**

**
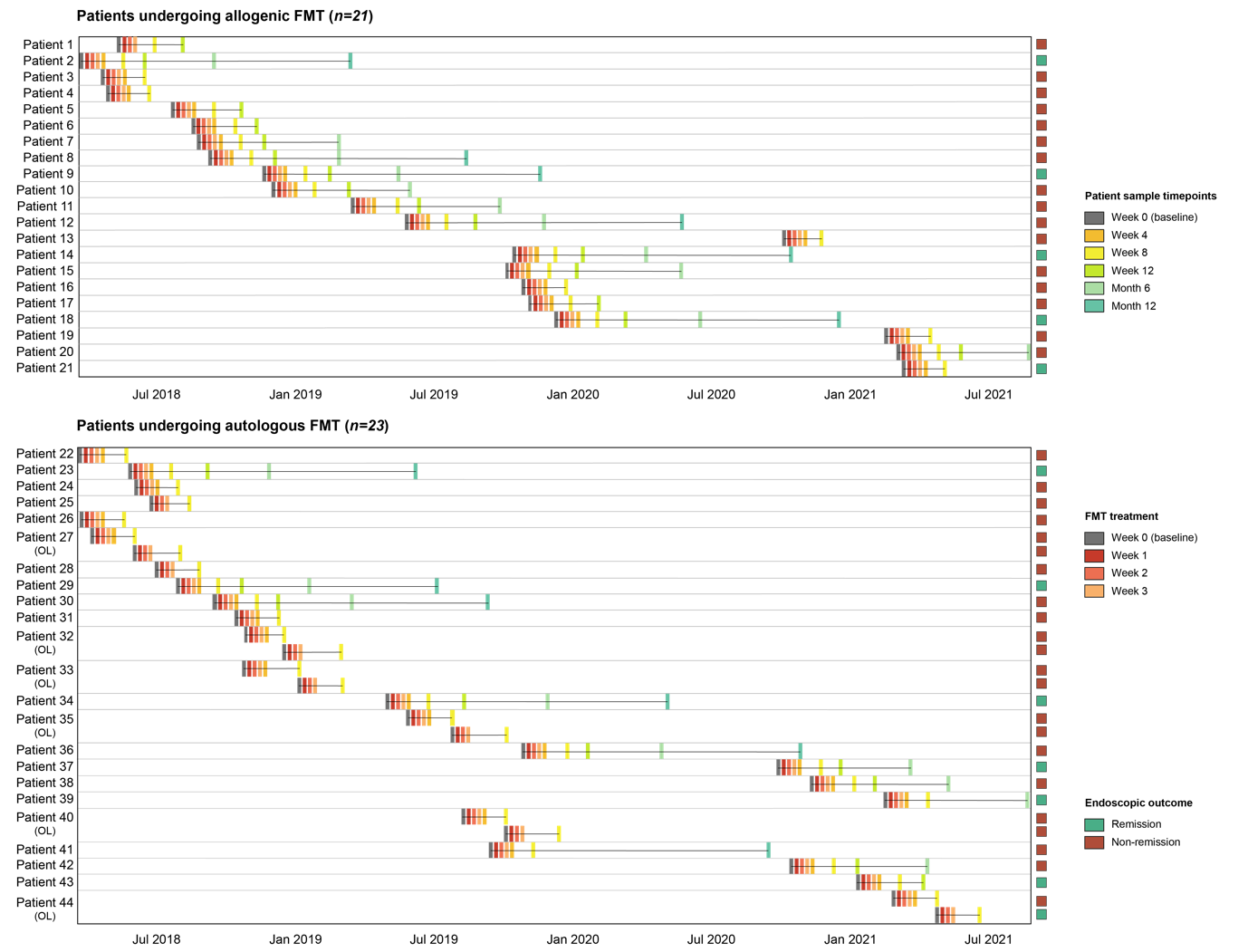
Extended Data Figure 1: A longitudinal randomized multicenter sham-controlled clinical trial of UC patients undergoing faecal microbiota transplants.** Study design of a longitudinal multicenter randomized sham-controlled UC cohort (UC patients=44) undergoing FMT treatment. Patients had active UC at baseline (pre-intervention, week 0) and underwent multiple rounds of treatment (week 0, week 1, week 2 and week 3) with either healthy donor FMTs (allogenic FMT) or sham FMTs (autologous FMT). At week 8 (post-intervention) patients were re-evaluated for the success of FMT treatment. In this study, a total of 320 faecal samples were selected from a combination of patients (N=44) and donors (N=31), given the availability of both baseline and week 8 samples. Among these samples, 197 samples were obtained specifically from patients (N=44) who underwent either healthy donor (N=21) or autologous FMTs (N=23). The remaining faecal samples (n=123) were collected directly from the donor FMT material administered to the patients which could come from either the healthy donor (samples=54, individuals=13) or autologous donors (samples=69, individuals=18). Abbreviations: Faecal microbiota transplant (FMT), Ulcerative colitis (UC) and open-label (OL).

 **Extended Data Figure 2: The acquisition and composition of high-quality viral metagenomes in the complete UC cohort. A,** Panel A provides an overview of the quality control steps performed on sequenced reads (raw reads=8.90 billion) and contigs (metaSPAdes contigs=22.6 million) within the UC cohort. Non-redundant contigs identified as prokaryotic or eukaryotic viruses were identified as “non-redundant viral contigs” (viral contigs=2224). Quality-controlled reads mapping to these non-redundant viral contigs were identified as viral reads (viral reads=3.16 billion), account for 35.5% of the raw reads. **B,** Panel B illustrates the distribution of various categories, including bacteria, viruses, unannotated sequences (dark matter), and others (archaea, protozoa and eukaryota), represented by the non-redundant contigs and by the quality-controlled mapped reads.

**
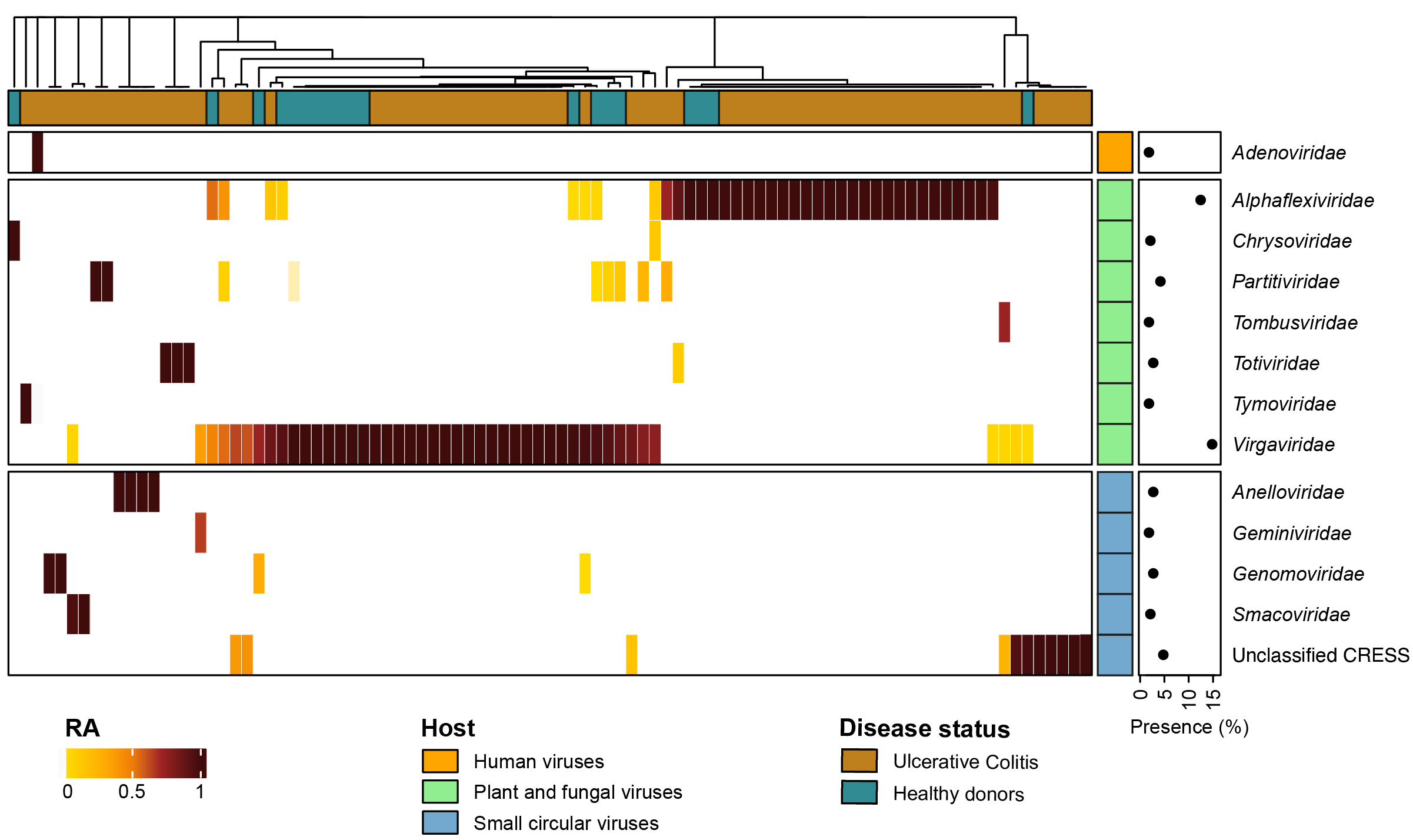
Extended Data Figure 3: The eukaryotic viral component in the complete UC cohort.** The heatmap represents the RA of eukaryotic viral families (AS ≥ 0.1) present in UC patients undergoing FMT (n=93, eukaryotic viral presence=30.6%). The eukaryotic viral families were categorized according to their host classification, including animal, plant/fungal and small circular viruses. This grouping enables a clear distinction between the different types of hosts that the viral families interact with. The disease status (healthy donor versus patient) of each individual from the corresponding sample is depicted on top of the heatmap. Abbreviations: Faecal microbiota transplant (FMT), Ulcerative colitis (UC), Relative abundance (RA) and alignment score (AS).

**
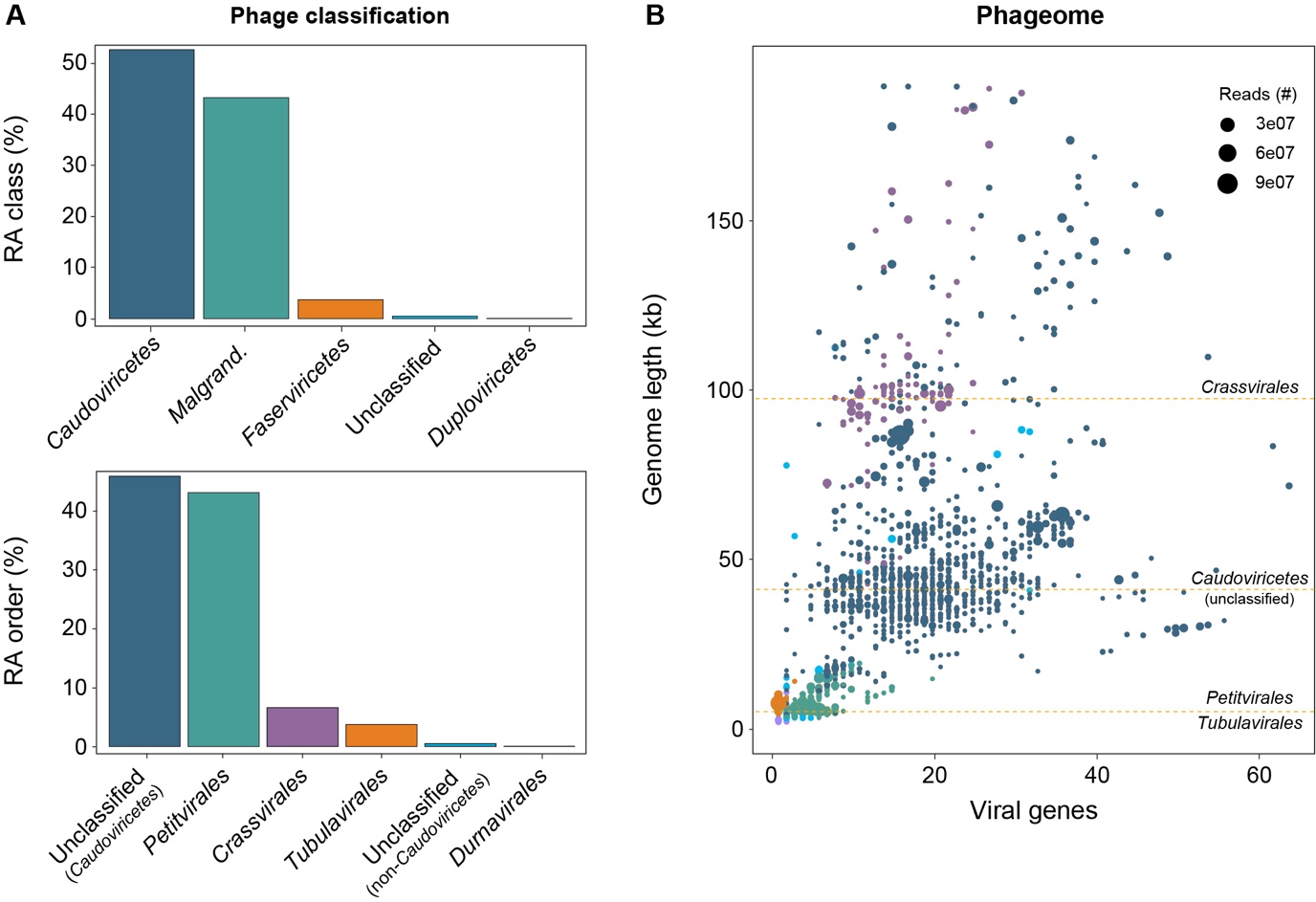
Extended Data Figure 4: Phageome characteristics in the complete UC cohort. A,** The relative abundance of classified (at the class and order taxonomic levels) and unclassified phages discovered in the complete UC cohort. **B,** A scatterplot showing the classification of the discovered phages genomes at order level stratified by the length (maximum of 200kb) and number of viral genes (maximum 65 viral genes). The number of viral-specific genes in this analysis were determined using CheckV. The median length for *Crassvirales* genomes is 98.4 kb (literature:97kb), unclassified *Caudoviricetes* genomes (literature:18kb-500kb) is 41.6 kb, *Petitvirales* is 6.02 kb (literature:5.3kb-6.1kb) and *Tubulavirales* is 5.57 kb (literature:5.5kb-10.6kb). The genomes size (in kilobases, kb) of phages mentioned in the literature were obtained from the International Committee for Taxonomy of viruses. Abbreviations: Relative abundance (RA) and ulcerative colitis (UC).

** Extended Data Figure 5: The relationship between viral gene density and *in silico* predicted bacterial hosts of discovered phages within the complete UC cohort.**

Scatterplot and barplot showing the major predicted bacterial hosts (at bacterial phyla level) of 1118 (51.2%) *Caudoviricetes* (unclassified), 770 (35.3%) *Petitvirales*, 103 (4.72%) *Crassvirales and* 48 (2.20%) *Tubulavirales* phage contigs and its relationship with viral gene density. False positive predictions are known to increase with shorter genome length and should be interpreted with caution (i.e., *Chlamydia*-infecting phages). Abbreviations: Ulcerative colitis (UC).
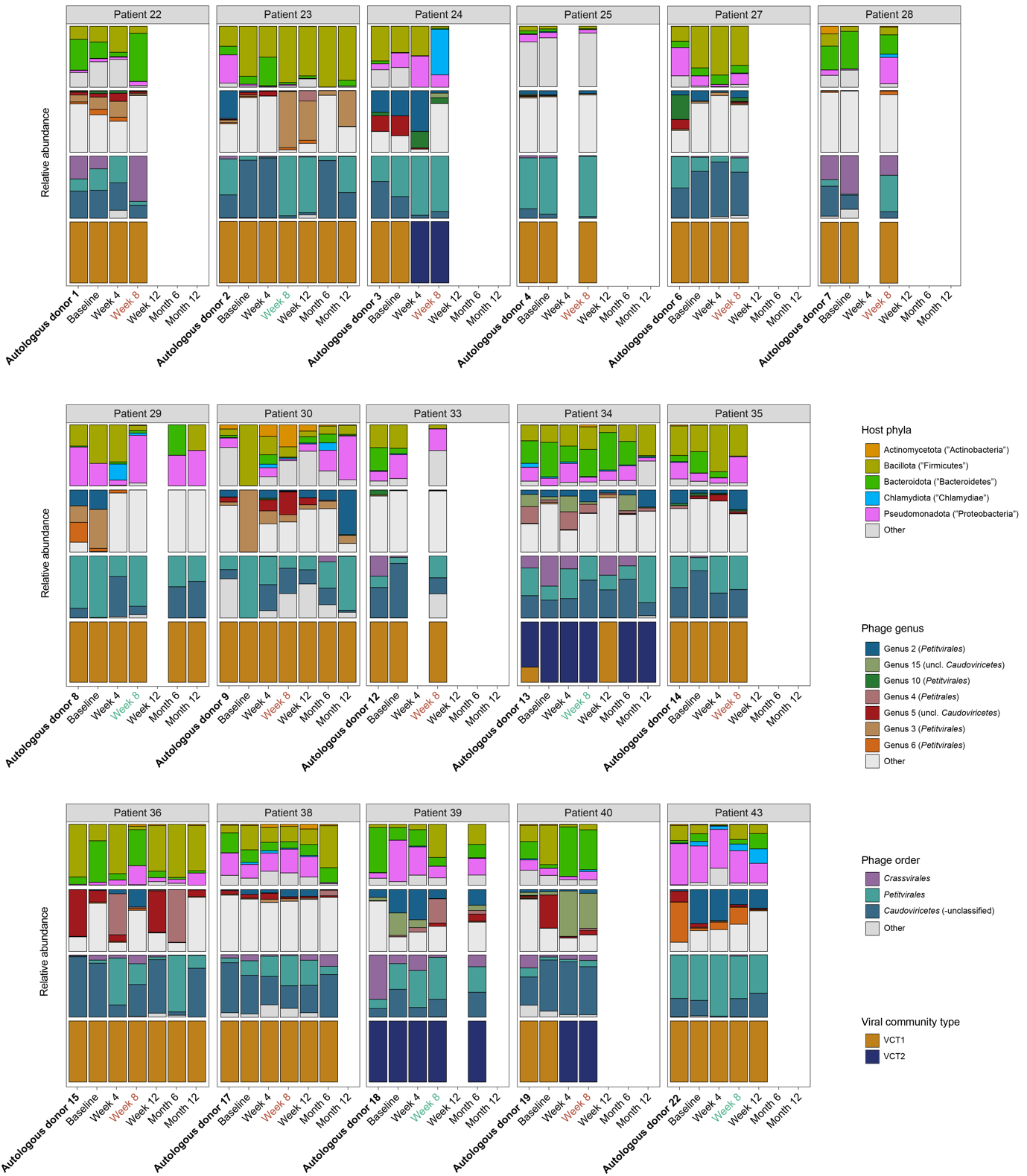
**Extended Data Figure 6: Summary of individual UC patients undergoing autologous faecal microbiota transplants.** Barplot showing the relative abundance of major (≥1% of reads) *in silico* predicted hosts, phage genera (≥15% prevalent), phage classes and viral community types, along a longitudinal axis for each UC patient undergoing autologous faecal microbiota transplantation. Each patient received a sequence of four autologous FMTs, collectively referred to as “Autologous donor x” (in bold), and were consistently derived from the same donor. Patients are shown who had available samples from the autologous donor, baseline (week 0) and week 8. The endoscopic outcomes at week 8 are represented as either remission (green) or non-remission (red). Abbreviation: Faecal microbiota transplantation (FMT) and ulcerative colitis (UC).

**
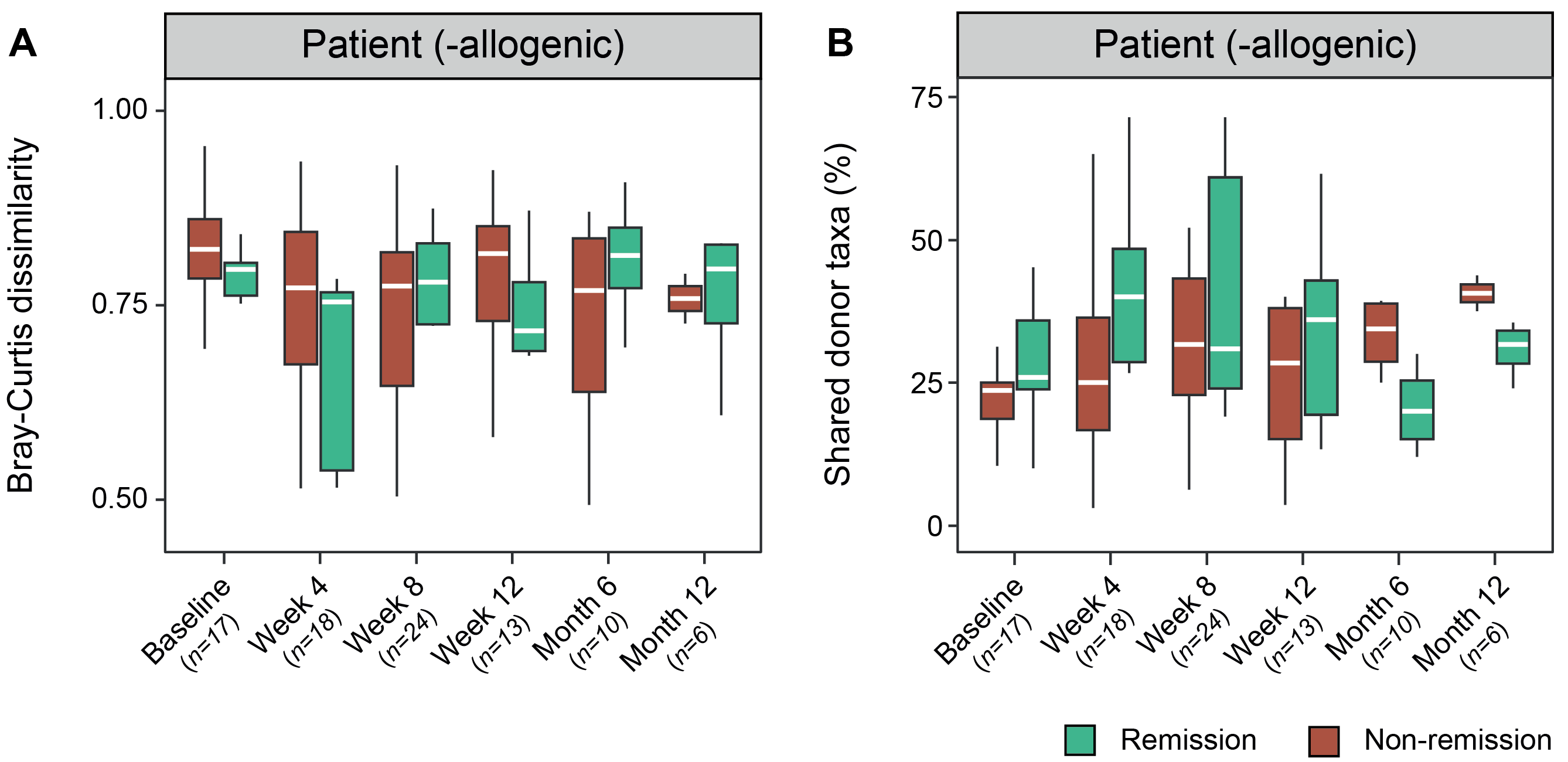
Extended Data Figure 7: The effect of endoscopic outcome on donor virome engraftment in UC patients undergoing allogenic FMTs. A,** Boxplot of inter-individual differences in the relative virome profiles (genus-like level, Hellinger transformation) between patients and their corresponding donor sample (LMM, AdjP>0.5) at different timepoints stratified by endoscopic outcome (remission/non-remission). **B,** Boxplot of the percentage of shared donor taxa between patients and its corresponding donor sample (LMM, AdjP>0.5) at different timepoints stratified by endoscopic outcome (remission/non-remission). Significant associations were determined after multiple testing (Benjamini-Hochberg method) by comparing each post-FMT to baseline timepoint and are represented by an asterisk (*). The aforementioned analyses include patient identifier as a grouping variable to account for the biological dependencies among multiple measurements within each patient. Abbreviations: Ulcerative colitis (UC) and linear mixed-effect model (LMM).

**Extended Data Figure 8: Identification of the ideal number of virome clusters or Dirichlet components within the complete UC cohort. A,** The ideal number of viral community types is identified based on the minimum Bayesian Information Criterion (BIC) of the complete UC cohort (*n=301*). The ideal number of viral community types is two. **B,** Barplot of the average relative abundance of highly prevalent viral genera (>15%) for each distinct viral community type. Multiple testing adjustment (Benjamini-Hochberg method) was performed and significant associations (AdjP<0.05) are represented by an asterisk (*). The aforementioned analyses include patient identifier as a grouping variable to account for the biological dependencies among multiple measurements within each patient. Abbreviations: Ulcerative colitis (UC) and viral community types (VCT).

**Extended Data Figure 9: The relationship between viral community types and *in silico* predicted bacterial host genera within the complete UC cohort.** Barplot showing the *in silico* predicted bacterial host genera of discovered phages (≥1% of reads) stratified according to viral community types (*n=301*, LMM, AdjP<0.5). Reliable host predictions for bacterial genera could be made for 26.6% of phages (84.8% for bacterial phyla). False positive predictions are known to increase with shorter genome length and should be interpreted with caution (i.e., *Chlamydia*-infecting phages). Multiple testing adjustment (Benjamini-Hochberg method) was performed and significant associations (AdjP<0.05) are represented by an asterisk (*). The aforementioned analyses include patient identifier as a grouping variable to account for the biological dependencies among multiple measurements within each patient. Abbreviations: Ulcerative colitis (UC) and viral community types (VCT) and linear mixed-effect model (LMM).
